# Addressing the ‘hypoxia paradox’ in severe COVID-19: literature review and report of four cases treated with erythropoietin analogues

**DOI:** 10.1186/s10020-021-00381-5

**Published:** 2021-09-26

**Authors:** Martin Begemann, Oliver Gross, Dominik Wincewicz, Rüdiger Hardeland, Vinicius Daguano Gastaldi, Eduard Vieta, Karin Weissenborn, Kamilla W. Miskowiak, Onnen Moerer, Hannelore Ehrenreich

**Affiliations:** 1 Clinical Neuroscience, Max Planck Institute of Experimental Medicine, Hermann-Rein-Str.3, 37075 Göttingen, Germany; 2 Department of Psychiatry and Psychotherapy, University Medical Center, Göttingen, Germany; 3 Department of Nephrology and Rheumatology, University Medical Center, Göttingen, Germany; 4 Hospital Clinic, Institute of Neuroscience, IDIBAPS, CIBERSAM, Barcelona, Spain; 5 Johann Friedrich Blumenbach Institute of Zoology & Anthropology, University of Göttingen, Göttingen, Germany; 6 Department of Neurology, Hannover Medical School, Hannover, Germany; 7 Psychiatric Centre Copenhagen, University Hospital, Rigshospitalet, Copenhagen, Denmark; 8 Department of Anaesthesiology, University Medical Center Göttingen, Göttingen, Germany

**Keywords:** Recombinant human EPO, Darbepoetin, Neuroprotection, Treatment, Signaling, Critical care, Outcome

## Abstract

**Background:**

Since fall 2019, SARS-CoV-2 spread world-wide, causing a major pandemic with estimated ~ 220 million subjects affected as of September 2021. Severe COVID-19 is associated with multiple organ failure, particularly of lung and kidney, but also grave neuropsychiatric manifestations. Overall mortality reaches > 2%. Vaccine development has thrived in thus far unreached dimensions and will be one prerequisite to terminate the pandemic. Despite intensive research, however, few treatment options for modifying COVID-19 course/outcome have emerged since the pandemic outbreak. Additionally, the substantial threat of serious downstream sequelae, called ‘long COVID’ and ‘neuroCOVID’, becomes increasingly evident.

**Main body of the abstract:**

Among candidates that were suggested but did not yet receive appropriate funding for clinical trials is recombinant human erythropoietin. Based on accumulating experimental and clinical evidence, erythropoietin is expected to (1) improve respiration/organ function, (2) counteract overshooting inflammation, (3) act sustainably neuroprotective/neuroregenerative. Recent counterintuitive findings of decreased serum erythropoietin levels in severe COVID-19 not only support a relative deficiency of erythropoietin in this condition, which can be therapeutically addressed, but also made us coin the term ‘hypoxia paradox’. As we review here, this paradox is likely due to uncoupling of physiological hypoxia signaling circuits, mediated by detrimental gene products of SARS-CoV-2 or unfavorable host responses, including microRNAs or dysfunctional mitochondria. Substitution of erythropoietin might overcome this ‘hypoxia paradox’ caused by deranged signaling and improve survival/functional status of COVID-19 patients and their long-term outcome. As supporting hints, embedded in this review, we present 4 male patients with severe COVID-19 and unfavorable prognosis, including predicted high lethality, who all profoundly improved upon treatment which included erythropoietin analogues.

**Short conclusion:**

Substitution of EPO may—among other beneficial EPO effects in severe COVID-19—circumvent downstream consequences of the ‘hypoxia paradox’. A double-blind, placebo-controlled, randomized clinical trial for proof-of-concept is warranted.

## Background

Since December 2019, a pandemic, COVID-19, caused by the severe acute respiratory syndrome coronavirus 2 (SARS-CoV-2) (Lu et al. 2020; Zhou et al. 2020; Zhu et al. 2020), approached 220 million confirmed cases and led to the death of ~ 4.5 million people worldwide, as of September 2021. Despite considerable success in developing vaccines (Haas et al. 2021; Sahin et al. 2021), COVID-19 keeps progressing around the globe with very limited advancement in the development of drugs for COVID-19 treatment (Batista et al. 2021; Kim et al. 2020; Kluge et al. 2021; WHO Solidarity Trial Consortium 2021; World Health Organization 2021a, b).

SARS-CoV-2 is a beta-coronavirus that infects cells via the angiotensin-converting enzyme 2 (ACE2) receptor. Multitropic by nature, it can in severe cases cause multi-organ failure, in particular of lung, heart, and kidney (Puelles et al. 2020). SARS-CoV-2 is often associated with a so-called cytokine storm (Fajgenbaum and June 2020; Mehta et al. 2020; Moore and June 2020), a hypercoagulable state (Bilaloglu et al. 2020; Klok et al. 2020; Levi et al. 2020), and induction of pronounced autoimmune reactions (Dotan et al. 2021; Halpert and Shoenfeld 2020; Wang et al. 2021). Via the olfactory mucosa, it penetrates to the central nervous system along the olfactory tract, appears to follow neuroanatomical structures, and affects predominantly the brainstem, including primary respiratory and cardiovascular control centers in the medulla oblongata (Matschke et al. 2020; Meinhardt et al. 2021). SARS-CoV-2 targets the blood–brain-barrier (BBB), affects endothelial cells (Buzhdygan et al. 2020; Rhea et al. 2021), and can lead to microvascular injury in the brain and spreading neuroinflammation of COVID-19 patients (Lee et al. 2021). Neurological manifestations or complications of COVID-19 disturb central and peripheral nervous system functions and structures, and have been shown in essentially all age groups (Kihira et al. 2020; LaRovere et al. 2021; Mao et al. 2020; Oxley et al. 2020; Paterson et al. 2020; Solomon 2021).

Persistent symptoms after recovery from the acute infection, referred to as post-acute or long COVID, as well as damage to the nervous system, inducing neuropsychiatric downstream syndromes, called neuroCOVID, may lead to significant long-term disability after survival of the acute phase (Al-Ramadan et al. 2021; Boldrini et al. 2021; Carfi et al. 2020; Davis et al. 2021; Huang et al. 2021; Iqbal et al. 2021; Nalbandian et al. 2021; Solomon et al. 2020; Sudre et al. 2021; Taquet et al. 2021; Townsend et al. 2020). Across brain cell types, COVID-19 perturbations overlap with those in chronic brain disorders and reside in genetic variants associated with cognition, schizophrenia, and depression (Yang et al. 2021). Due to the recognized neuroprotective, procognitive and neuroregenerative capacities of erythropoietin (EPO) in exactly these conditions, this very recent report strongly supports its application for preventing neuroCOVID, as outlined below. In general, host genetic factors influence the severity of the COVID-19 course (Kuo et al. 2020; Severe Covid-19 GWAS Group 2020). Among the risk genes is for instance the APOE4 allele that affects BBB integrity (Hammer et al. 2014; Kuo et al. 2020; Masoli et al. 2021; Montagne et al. 2020).

Despite intensive research within the last year, only few treatments for COVID-19 are currently available (Kim et al. 2020; Kluge et al. 2021; WHO Solidarity Trial Consortium 2021), and to date, no effective measures are known to improve conditions of long COVID or neuroCOVID (Llach and Vieta 2021). This scarcity of options for the management of COVID-19 and the emergence of ‘immune escape’ against established vaccines plus ‘breakthrough infections’ (Birhane et al. 2021; Edara et al. 2021; Garcia-Beltran et al. 2021; Li et al. 2021; Plante et al. 2021; Prevost and Finzi 2021; Thorne et al. 2021; Wall et al. 2021; Zhou et al. 2021), reducing the hope of a speedy termination of the pandemic, call for new strategies to treat COVID-19. Based on the known neuroprotective and anti-inflammatory features of EPO, and its proven efficacy and excellent tolerability in different neuropsychiatric conditions (Ehrenreich et al. 2020; Miskowiak et al. 2021; Sargin et al. 2010; Sirén et al. 2009), we proposed the use of recombinant human (rh) EPO for the treatment of severely affected COVID-19 patients. The sketched double-blind, placebo-controlled, randomized clinical trial (Ehrenreich et al. 2020), has not yet received appropriate funding. However, our proposal is now strongly reinforced by further post-mortem data on COVID-19 (Yang et al. 2021). Here we present four cases of severe COVID-19 and unfavorable prognosis, that received EPO analogues. These were the only cases we became aware of except for a published case report (Hadadi et al. 2020). We report the disease course of these male patients upon treatment and discuss possible mechanisms contributing to their beneficial outcome.

## Cases

The 4 patients (individual synopses presented in Table 1) were all men in the range of 58 to 80 years, who presented with dyspnea, malaise, and other nonspecific signs of viral infection, and were confirmed by nasopharyngeal swab and PCR to be positive for SARS-CoV-2. All patients progressed swiftly to respiratory failure, were critically ill, hospitalized and treated in intensive care units (ICUs) for variable length of time. The duration of severe hypoxia, requiring oxygen supplementation, amounted to only 3 days in case 1—under the immediately started rhEPO application as compassionate use approach, in absence of any approved treatment for severe COVID-19. This unusually fast improvement may at least hypothetically be attributable to EPO. The underlying beneficial effects likely go beyond erythropoiesis and include counteraction of the complex pathophysiology of COVID-19 as delineated in the present paper and previous reviews on EPO in COVID-19 (Ehrenreich et al. 2020; Sahebnasagh et al. 2020). The 3 other patients (cases 2–4) developed severe acute respiratory distress syndrome (ARDS), clinically reflected by the need for invasive ventilatory settings, and additional prone positioning. Acute renal injury and cardio-circulatory deterioration were swiftly prominent in these patients and led to an instant treatment with EPO analogues. Cases 2 and 4 needed renal replacement therapy. Cases 2–4 were on anticoagulation treatment as is the standard for COVID-19 in German intensive care units, at least the low molecular weight heparin for prophylaxis of venous thrombosis. All 4 patients of our case report series were anemic, additionally underlining their severe disease state (Taneri et al. 2020). None of them received blood transfusions. The iron status of all remained within the normal range or close to normal (transferrin saturation 12–31%; normal range 16–45%). Other laboratory values, as much as available for all four cases, were further consistent with a severe course of COVID-19 with hypoxia and highly elevated inflammatory markers in the sense of a cytokine storm (Table 1). All 4 patients received injections of rhEPO analogues, according to conventional dosing for anemia treatment in nephrology (around 2000–4000 IU per injection), and recovered either during hospitalization (Case 1; the least severe of our cases) or during rehabilitation care (Cases 2–4).

**Table 1:** Synopsis of 4 male COVID-19 patients treated with EPO analogues

	Case 1	Case 2	Case 3	Case 4
**Age** (years)	68	59	80	58
**Gender**	Male	Male	Male	Male
**Time/place of SARS-CoV-2 contact**	March 2020, Poland	March 2020, Austria	February 2021, Germany	February 2021, Germany
**Premedical history**	Chronic kidney disease (stage 2) Arterial hypertension	Intermittent atrial fibrillation Arterial hypertension Past heavy smoker	Chronic kidney disease (stage 2) Chronic obstructive pulmonary disease Arterial hypertension Pulmonary embolism 2016 Past heavy smoker	Arterial hypertension Adipositas Metabolic syndrome
**Presenting symptoms**	Cough, dyspnea, fever	Unspecific malaise, dyspnea	Unspecific malaise, dry cough, headache, diarrhea	Unspecific malaise, dyspnea, diarrhea
**Course, complications and treatment**	Progression to **respiratory failure** with hypoxemia and normocapnia Transfer to ICU with nasal O_2_ insufflation Delivery of oxygen through face mask (initial flow 6 L/min, finally reduced to 4 L/min to target SPO_2_ of 90–92% Supportive treatment only	Progression to **respiratory failure** Transfer to ICU with **ARDS** (mechanical ventilation for 24 days; intermittent prone position; max FiO_2_ 100%, max inspiratory pressure 30 cmH_2_O, max PEEP 14 cmH_2_O) **AKI** with renal replacement therapy for 42 days Critical care/treatment with: • Anticoagulation with heparin/argatroban •Prednisolon pulse therapy • Toculizumab infusion • Antiviral therapy: Lopinavir/Ri-tonavir for 2 weeks at start of hospital stay, hydrochloroquine **Critical illness polyneuropathy**	Progression to **respiratory failure** Transfer to ICU with **ARDS** (high-flow oxygen therapy HFNC and intermittent non-invasive mechanical ventilation for 21 days, intermittent prone position; max FiO_2_ 85%, max. pressure support 6 cmH_2_O, max PEEP 8 cmH_2_O) **AKI** without need for renal replacement therapy (minimal eGFR 28 ml/min) Critical care/treatment with: • Anticoagulation with heparin • Prednisolon pulse therapy • Antiviral therapy: none **Critical illness polyneuropathy**	Progression to **respiratory failure** Transfer to ICU with **ARDS** (mechanical ventilation for 17 days, intermittent prone position; max FiO_2_ 100%, max inspiratory pressure 26 cmH_2_O, max PEEP 12 cmH_2_O) **AKI** with renal replacement therapy for 6 days Critical care/treatment with: • Anticoagulation: heparin • Prednisolon pulse therapy • Antiviral therapy: Camostat for 10 days **Critical illness polyneuropathy**
**EPO analogue Treatment** (started within the first 2 days of ICU admission)	Recombinant human EPO (Epoetin beta, NeoRecormon- Roche 20 IU/kg body weight, every 12 h; 5 infusions)	Darbepoetin alfa (40 µg sc. once weekly, Aranesp-Amgen) for 5 weeks	Darbepoietin alfa (40 µg sc. once weekly, Aranesp-Amgen)—for 4 weeks	Darbepoietin alfa (40 µg sc. once weekly, Aranesp -Amgen) for 3 weeks
**EPO equivalent dose in total**	100 µg (10,000 IU)	200 µg (20,000 IU)	160 µg (16,000 IU)	120 µg (12,000 IU)
**Outcome**	Discharge from ICU after 72 h; patient remained hospitalized for additional 3 weeks Respiratory: complete recovery	Discharge after 8 weeks ICU stay, rehabilitation care 3 weeks Respiratory: complete recovery Renal: complete recovery Neurological: complete recovery	Discharge after 44 days with 22 days ICU stay Respiratory: recovery Renal: recovery Neurological: partial recovery, remaining general weakness	Discharge after 31 days with 19 days ICU stay Respiratory: complete recovery Renal: complete recovery Neurological: complete recovery
**Laboratory parameters** (inflammation markers)	**CRP** max. 79.9 mg/l (normal < 5.0 mg/l)	**CRP** max. 119.6 mg/l (normal < 5.0 mg/l) **IL-6**: 259–775 pg/ml (normal < 7.0 pg/ml) **s-Interleukin 2 R** (IU/ml): 3359–6189 (normal range 223–710); **d-Dimers**: max. 7.11 mg/l (normal < 0.5 mg/l) **Fibrinogen**: max. 936 mg/dl (normal range 200–393 mg/dl)	**CRP** max. 210.0 mg/l (normal < 5.0 mg/l) **IL-6**: 68 pg/ml (normal < 7.0 pg/ml) **s-Interleukin 2 R**: N/A **d-Dimers**: max. 6.64 mg/l (normal < 0.5 mg/l) **Fibrinogen**: max. 927 mg/dl (normal range 200–393 mg/dl)	**CRP** max. 119.9 mg/l (normal < 5.0 mg/l) **IL-6**: 18 pg/ml (normal < 7.0 pg/ml) **s-Interleukin 2 R** (IU/ml): 3002–3038 (normal range 223–710) **d-Dimers**: max. 17.42 mg/l (normal < 0.5 mg/l) **Fibrinogen**: max. 661 mg/dl (normal range 200–393 mg/dl)
**Laboratory parameters** Hematocrit (Hct) Hemoglobin (Hb)	**Prior to EPO:** Hb 9.6 g/dl; Hct 28.4% **Discharge from ICU (EPO subsequently stopped):** Hb 10.1 g/dl; Hct 29.9%	**Prior to EPO:** Hb 8.1 g/dl; Hct 22.7% **Discharge from ICU (with EPO):** Hb 8.7 g/dl; Hct 26.0% **EPO discontinued at:** Hb11g/dl; Hct 35%	**Prior to EPO:** Hb 7.9 g/dl; Hct 23.1% **Discharge from ICU (with EPO):** Hb 8.1 g/dl; Hct 24.9% **EPO discontinued at:** Hb > 11 g/dl; Hct ca. 35%	**Prior to EPO:** Hb 9.5 g/dl; Hct 27.9%, **Discharge from ICU (with EPO):** Hb 11.7 g/dl; Hct 34.5% **EPO discontinued at:** Hb 12 g/dl

Bold indicates common symptoms/measurements shared between the cases

*AKI* acute kidney injury, *ARDS* acute respiratory distress syndrome, *HFNC* high flow nasal canula, *ICU* intensive care unit, *max* maximal, *N/A* not available

## Discussion

We recently advocated the use of rhEPO for supportive treatment of severe COVID-19, provided a comprehensive review backing this idea, and published the design of a clinical trial (Ehrenreich et al. 2020). So far, we did not succeed in obtaining the necessary funding for a clinical study. Since rhEPO has been approved for decades as well-tolerated and safe anemia drug, funding agencies send applicants for clinical rhEPO projects to industry or at least request industry to take a share in the costs of a clinical trial. Upon patent expiration in 2008, however, industry faces competition for the highly lucrative anemia market by biosimilar producers and fears off-label use and emergence of new side effects, thus unfortunately does not welcome clinical research on further rhEPO indications. Hence, at this point, we can only present as further supporting hints for our concept, 4 male patients with severe COVID-19 who were treated with EPO analogues. These were the only COVID-19 cases who received EPO analogues we became aware of and had access to. In all cases, EPO was well tolerated and associated with remarkable recovery despite severe COVID-19 with multiple critical complications, including respiratory and renal failure, documented cytokine storm, and critical illness polyneuropathy. Additional factors in our cases, known to be associated with unfavorable prognosis in hospitalized patients, were older age, male gender, and their comorbidities (Becher and Frerichs 2020).

We had originally considered for our small case series a ‘center control group’, however, had to surrender this plan because in our hospital, sufficient numbers of adequate male patients with comparable age and disease severity would not have been available for a convincing ‘twin-matching’ (which would require at least 1:3 or 1:4, i.e. N = 12–16 suitable ‘statistical twins’). Instead, and also to avoid an impression of ‘cherry picking’ controls post-hoc, we cite outcomes of highly representative German surveys, e.g. on 10,021 patients with COVID-19 admitted to 920 German hospitals (Karagiannidis et al. 2020). In-hospital mortality in mechanically ventilated patients requiring dialysis was here 73% (342 of 469). In the respective observational time period of our cases, the mortality of mechanically ventilated COVID-19 patients in ICUs in Germany remained at 50% across age groups even with introduction of additional therapeutic measures like early corticosteroids/dexamethasone or IL-6 blockade (Karagiannidis et al. 2021; Kluge et al. 2021; Wilkinson 2020). Clinical course and laboratory values in cases 2–4 showed dramatic increases in IL-6, often as part of the cytokine storm and predictor of poor survival (Chang et al. 2021; Chen et al. 2020; Mehta et al. 2020; Moore and June 2020; Tan et al. 2020; Webb et al. 2020).

In striking contrast to the very poor prognosis is the outcome with full recovery of all patients. The here presented four cases are consistent with a prior case report of an 80 year-old man with multiple medical comorbidities who recovered completely after EPO treatment (Hadadi et al. 2020). The total EPO equivalent dose that this case and our 4 patients received, ranged between 10,000 and 20,000 IU (100–200 µg) over 1–5 weeks, corresponding to the conventional dosing for anemia treatment in nephrology (around 2000–4000 IU per injection). These doses are low as compared to those needed for improving cognition and motor performance in neuropsychiatric indications like multiple sclerosis, schizophrenia or affective disorders (Ehrenreich et al. 2007a, b; Miskowiak et al. 2014a, b) or for reducing progressive brain matter loss in these conditions (Miskowiak et al. 2015; Wüstenberg et al. 2011). Although the presence of a slightly compromised BBB in some patients suffering from these neuropsychiatric diseases is well established and allows a higher quantity of EPO to reach the brain (Ehrenreich et al. 2004), it is unlikely equivalent to the massive BBB breakdown expected in severe COVID-19 with the typical cytokine storm. This may explain why in COVID-19 comparably low doses seem sufficient also for improving neurological sequelae which was not the case in e.g. multiple sclerosis (Ehrenreich et al. 2007a). Nevertheless, in a potential clinical trial, investigating EPO treatment for improving outcome of severe COVID-19 patients (Ehrenreich et al. 2020), and particularly for preventing neuroCOVID and long COVID (Llach and Vieta 2021), at least 10× higher doses may have to be tested—perhaps as parallel study arm—to disclose the optimal amount required for these conditions.

### Safety issues

Overall, in critically ill patients, EPO is safe and probably efficient, as summarized in recent meta-analyses (Litton et al. 2019; Mesgarpour et al. 2017). Nevertheless, employing EPO in severe COVID-19 for improving acute and downstream outcome (long COVID), requires watchful and comprehensive safety management as much as in all other EPO indications and, in particular, when using high doses of EPO to obtain sufficient levels also in brain (Bartels et al. 2008). Careful observation and follow-up at all times is mandatory, including clinical examination (blood pressure monitoring etc.) as well as routine laboratory screening (hematocrit, hemoglobin, thrombocyte counts etc.) during the period of EPO application to prevent augmentation of prothrombotic constellations in addition to the known hypercoagulable state in COVID-19 (Bilaloglu et al. 2020; Klok et al. 2020; Levi et al. 2020). Hematocrit/hemoglobin have to stay within clearly defined limits. Although the necessity of blood lettings in COVID-19 is expectedly extremely low due to the here reviewed reduced production of and/or response to EPO, in the unlikely situation of hematocrit increase towards the upper limit, blood letting should be initiated. Along the same lines, no iron substitution is allowed in this indication at any time, as it could possibly induce undesired ‘side effects’ in non-anemic COVID-19 patients. Iron substitution, as performed in studies targeting pure anemia, acts pro-inflammatory and may push hematopoiesis, even though expectedly weaker in the inflammatory environment of COVID-19. The fact that EPO treatment leads to temporary shifts in iron stores, and upon longterm application causes a picture similar to that of true iron deficiency, as reported in chronic progressive multiple sclerosis (Ehrenreich et al. 2007a), might even provide additional benefit for COVID-19 patients, adding to the panel of protective effects of this growth factor. Notably, also iron chelators have been proposed for treatment of COVID-19 (Carota et al. 2021).

### Hypothetical explanations of the ‘hypoxia paradox’

Surprisingly, recent reports showed decreased serum EPO levels in patients with severe COVID-19 (Viruez-Soto et al. 2021; Yagci et al. 2021). In the context of hypoxia, as experienced in this condition, we would rather have expected increased levels of EPO as a hypoxia-inducible growth factor (Brines and Cerami 2005; Jelkmann 1992; Krantz 1991). This phenomenon, which we coined ‘hypoxia paradox’, likely contributes to unfavorable outcomes in COVID-19. Along these lines of thought, people living at high-altitude have elevated levels of serum EPO and are therefore believed to be better protected from a severe course of COVID-19 (Arias-Reyes et al. 2020, 2021; Beall 2007; Jaramillo et al. 2021; Soliz et al. 2020; Viruez-Soto et al. 2021; Zubieta-Calleja et al. 2020). In our patients, serum EPO levels were not available, as EPO determinations are not part of any clinical laboratory routine, but an assumption of diminished levels would perfectly fit with their documented anemia prior to EPO treatment (Table 1). Interestingly, a recent systematic review and meta-analysis also reported that severe COVID-19 cases had lower hemoglobin levels compared to moderate cases (Taneri et al. 2020). However, even if baseline serum EPO concentrations in our patients had been normal, not lowered (which we do not know), this would not abrogate potential beneficial effects of exogenous rhEPO administration.

**Fig. 1:**
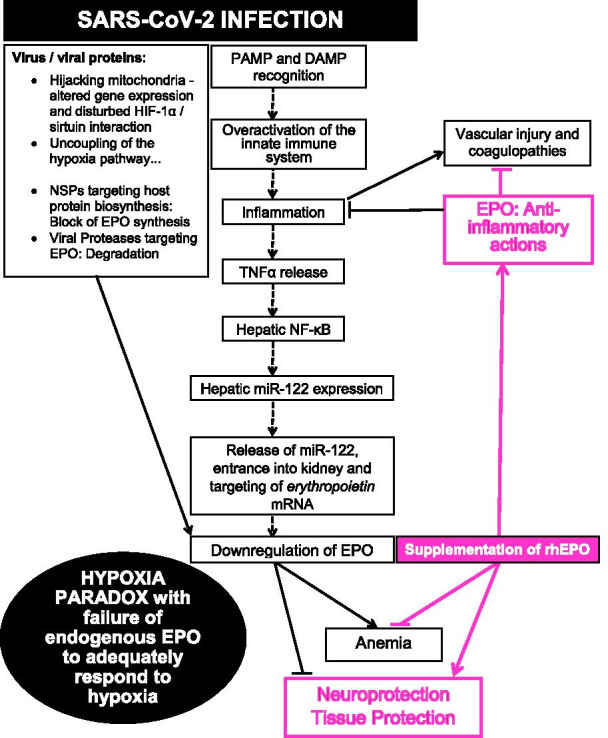
**Suppression of EPO expression as a consequence of SARS-CoV2 infection and the rationale for rhEPO supplementation.** Putative action of viral nonstructural proteins (NSP) and viral proteases on host protein biosynthesis and stability of host proteins (see text for references and details). Failure of EPO response to hypoxia may result in anemia and enhanced tissue/brain/neural injury. External EPO or EPO biosimilars are expected to combat anemia, reconstitute neuroprotection and tissue protection and mediate beneficial immune modulation. PAMP (pathogen-associated molecular pattern) and DAMP (damage-associated molecular pattern) recognition can induce primary and secondary cytokine storms, respectively. EPO suppression by TNFα and by TNFα/NF-κB-mediated induction of miR-122 has been demonstrated under various inflammatory conditions (Cluzeau et al. 2017; Rivkin et al. 2016). Anti-inflammatory actions, vascular protection and beneficial effects of EPO on respiratory functions have been reviewed in detail earlier (Ehrenreich et al. 2020)

The apparent ‘hypoxia paradox’ indicates dysfunctional signaling of the hypoxia circuitry required for the induction of EPO. The molecular mechanisms behind this ‘hypoxia paradox’ are still widely unknown. Several hypotheses that will have to be experimentally addressed in the future are listed here and sketched in Fig. 1:

1. Mitochondrial dysfunction: SARS-CoV-2 obviously hijacks host mitochondria in COVID-19 pathogenesis (Singh et al. 2020). By SARS-CoV-2 protein interaction map (Gordon et al. 2020) or by combining an RNA-centric approach and functional CRISPR screens, physical and functional connection between SARS-CoV-2 and mitochondria has been demonstrated, highlighting this organelle as a general platform for antiviral activity (Flynn et al. 2021). Moreover, altered expression of mitochondrial genes has been demonstrated by scRNAseq in a primate model of Corona infection (Speranza et al. 2021), and SARS-CoV-2 downregulated nuclear-encoded mitochondrial genes related to cellular respiration and complex I (Miller et al. 2021). Mitochondrial dysfunction has further been reported in the context of metabolic programs that define dysfunctional immune responses in severe COVID-19 (Thompson et al. 2021).
  Hypoxia signaling via HIF-1α and dioxygenase enzymes, prolyl hydroxylases 1,2,3 (PHD1,2,3), requires intact mitochondria. In COVID-19 sepsis, mitochondria become dysfunctional through a disturbed HIF-1α/sirtuin pathway (Shenoy 2020). Mitochondrial function and HIF are intimately interconnected to regulate each other (Tormos and Chandel 2010), and their impairment may well contribute to uncoupling of the hypoxia pathway. Tissue proteomic studies in turn showed the induction of hypoxia pathways in different organs of patients who deceased from COVID-19, but did not list EPO (Nie et al. 2021). This is perhaps less surprising, considering that due to its very low expression, mRNA of EPO regularly escapes single cell mRNA-seq analysis, a known dropout effect of this methodology. We note that EPO is an extremely potent hypoxia-inducible factor, locally effective in auto-paracrine fashion at femtomol concentrations (Butt et al. 2021; Wakhloo et al. 2020). In the situation of COVID-19, however, the low expression is likely further inhibited as a result of the uncoupled hypoxia pathway.
2. Inflammation and biosynthesis of EPO: Inflammatory processes induce the expression of microRNA122 that targets an evolutionary well-preserved seed site in the 3ʹUTR of *EPO* leading to reduced biosynthesis of EPO in the kidney (Rivkin et al. 2016). Moreover, proinflammatory proteins S100A9 and TNFα suppress EPO expression e.g. in myelodysplastic syndrome (Cluzeau et al. 2017). EPO biosynthesis might be further inhibited by viral gene products like NSP1 (nonstructural protein 1) which suppresses production of a whole array of host proteins (Yuan et al. 2020). This could also occur via derangement of host signaling circuitries as shown for structural proteins of SARS-CoV-2 (Jakhmola et al. 2021).
3. Proteolysis and degradation of EPO: The viral serine proteases such as NSP3 and/or NSP5 (Dai et al. 2020; Osipiuk et al. 2021; Zhang et al. 2020) might target EPO as well and lead to its proteolysis. Interestingly, recently identified inhibitors against the SARS-CoV-2 encoded main protease have been suggested as a treatment option for COVID-19 (Günther et al. 2021). In addition, host proteases that are induced in severe COVID-19 might proteolyse EPO.
4. EPO efficiency: In addition to its reduced production or enhanced degradation, leading to low circulating levels, the efficiency of EPO is likely reduced upon inflammation, as long known for its hematopoietic effects. This relative hyporesponsiveness of the haematopoietic system to EPO in systemic inflammatory conditions has been explained by altered cytokine patterns that modulate the bone marrow response to EPO (Kwack and Balakrishnan 2006). This may be partially overcome by higher doses and does not necessarily reflect on extrahematopoietic effectiveness of EPO (Ehrenreich et al. 2007a).

## Conclusions

Substitution of EPO appears as a rational strategy (Fig. 1) to circumvent downstream consequences of the ‘hypoxia paradox’, including dysfunctional synthesis or accelerated inactivation of endogenous EPO, in addition to the likely beneficial pharmacological profile of EPO applications pointed out earlier (Ehrenreich et al. 2020; Leventhal et al. 2020; Sahebnasagh et al. 2020). The obvious limitation of the present work is that case reports can only serve as first hints and ultimately require rigorous confirmation in a clinically controlled setting. An appropriate double-blind, placebo-controlled, randomized clinical trial is prerequisite for claiming any beneficial effects of rhEPO or biosimilars for the treatment of severely affected COVID-19 patients as well as for preventing COVID-19 downstream sequelae, long COVID and neuroCOVID.

## Data Availability

Not applicable.
